# Regression of Acoustic Tumor After Chemotherapy for Ovarian Cancer in a Patient With a Breast Cancer Susceptibility Gene 1 (BRCA1) Germline Mutation

**DOI:** 10.7759/cureus.35917

**Published:** 2023-03-08

**Authors:** Anna Takeda, Masahito Kobayashi, Kosei Hasegawa, Takamitsu Fujimaki

**Affiliations:** 1 Neurosurgery, Saitama Medical University Hospital, Saitama, JPN; 2 Gynecologic Oncology, Saitama Medical University International Medical Center, Saitama, JPN

**Keywords:** brca1, breast cancer susceptibility gene, acoustic tumor, olaparib, brca1 germline mutation, ovarian cancer, case report, vestibular schwannoma

## Abstract

We report the case of an adult woman who developed ovarian cancer during a follow-up for vestibular schwannoma. Volume reduction of the schwannoma was observed after chemotherapy for ovarian cancer. After ovarian cancer had been diagnosed, the patient was found to have a germline mutation of breast cancer susceptibility gene 1 (BRCA1). This is the first reported case of vestibular schwannoma in a patient with a germline mutation of BRCA1 and the first documented example of chemotherapy including olaparib to have shown efficacy for schwannoma.

## Introduction

Vestibular schwannoma often shows a slow growth rate, and tumors that are not large with only minimal neurological symptoms are often not followed up intensively with periodic magnetic resonance imaging (MRI) scans [1]. Though it is often quoted to affect about one in 100,000 people, a recent global systematic review estimated the lifetime prevalence of developing vestibular schwannoma to be more than one in 500 people [2]. When treatment is needed, surgery and stereotactic radiosurgery are the established treatment options [3], as reports of drug treatments with proven efficacy are limited, except for tumors associated with neurofibromatosis type 2 (NF2), which are sometimes treated with bevacizumab or lapatinib [4].

Here we report an adult female patient with an acoustic tumor that was presumed to be vestibular schwannoma who later developed ovarian cancer with a germline mutation of breast cancer susceptibility gene 1 (BRCA1) and whose acoustic tumor regressed during chemotherapy for ovarian cancer.

Germline mutations in BRCA1 can cause cancers of the breast, ovary, prostate, and pancreas [5-7]. Another epidemiological study suggested a relationship between a family history of breast cancer and an elevated risk of meningioma, but none for vestibular schwannoma [8].

This case is important because it shows that vestibular schwannoma may occur in patients with germline mutations of BRCA1 and that chemotherapy such as olaparib might be effective for vestibular schwannoma.

## Case presentation

**Figure 1:**
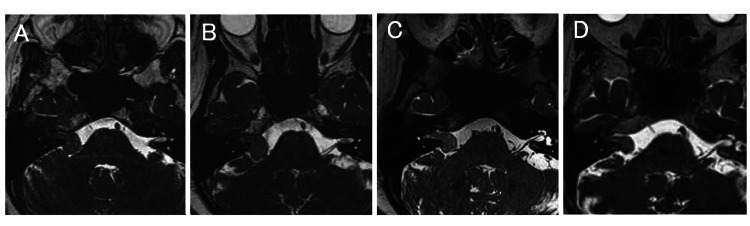
Serial constructive interference in steady state (CISS) MRI during the disease course. (A) March 18, 2013; (B) April 1, 2017; (C) March 20, 2018; (D) April 20, 2021

A 60-year-old woman with no significant medical history presented to our hospital in March 2013 with right-sided hearing loss that had become evident over the past five years. She also complained of tinnitus and vertigo. Standard pure-tone audiometry revealed that the mean hearing threshold level calculated by the one-fourth method on the right side was 80 dB (the left side was 21.3 dB) at a frequency of over 500 Hz. Speech discrimination on the right side was 25% at a speech level of 100 dB, contrary to the left side, which was 100% at 60 dB. Nystagmus was not observed in the caloric test on the right side, indicating severe dysfunction of the semicircular canal. Furthermore, balance tests indicated impairment of both upper and lower vestibular functions on the right side. An MRI demonstrated a tumor with a maximum diameter of approximately 20 mm protruding from the right internal auditory canal into the cerebellopontine angle (Figure 1A). Based on the clinical symptoms and imaging findings, a clinical diagnosis of vestibular schwannoma was made. Since the tumor was relatively small and did not compress the brainstem or cerebellum, the patient was scheduled for regular outpatient follow-up.

**Figure 2:**
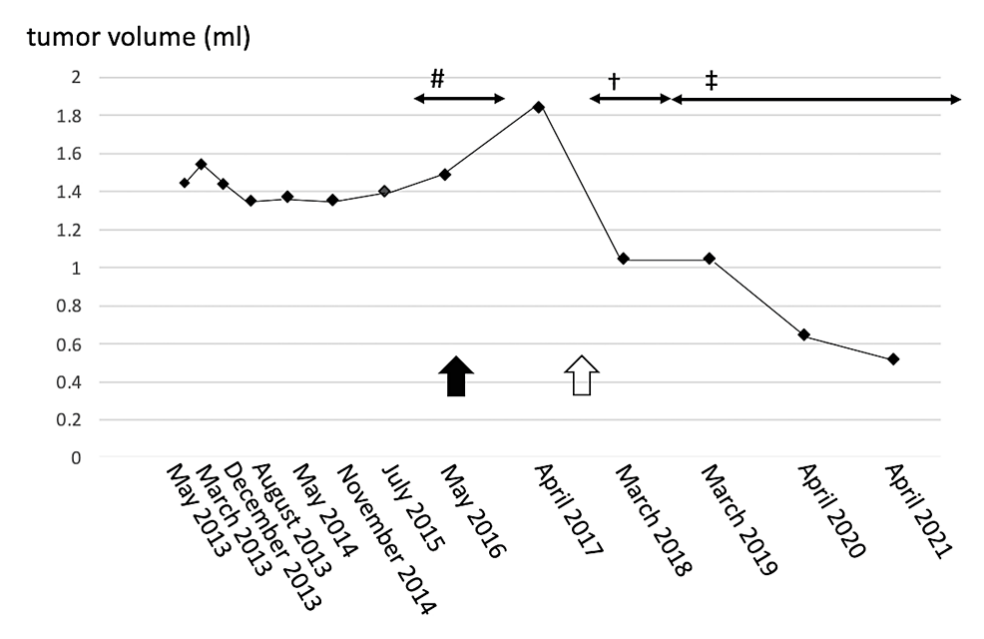
Changes in cerebellopontine angle tumor volume over time and their relationship to therapy for ovarian cancer. The tumor was slowly growing until April 2017, but MRI after the initiation of carboplatin and docetaxel chemotherapy showed a decrease in tumor size, and the tumor continued to shrink during further olaparib chemotherapy. #: carboplatin and paclitaxel; †: carboplatin and docetaxel; ‡: olararib Black arrow: resection of the abdominal tumor; white arrow: ovarian cancer recurrence

Two years later, in September 2015, she visited the gynecologic oncology department of the International Medical Center, Saitama Medical University, with a complaint of abdominal distention, and stage IIIc ovarian cancer was diagnosed, with seeding of the peritoneum and diaphragm. Carboplatin and paclitaxel (dose-dense paclitaxel 80 mg/m2 and carboplatin) were administered from November 2015 to March 2016, followed by the resection of the ovarian tumor in April 2016. Due to adverse events, including myelosuppression and skin symptoms, the target area under the concentration-time curve (AUC) of carboplatin was reduced from 6 to 5 mg/min/ml, and the sixth to eleventh courses of dose-dense carboplatin and paclitaxel were administered from May to December 2016. At this point, the abdominal tumor disappeared from the images, and a complete response was obtained. However, an abdominal CT scan in November 2017 revealed a recurrence of ovarian cancer in the form of peritoneal dissemination. As a peripheral blood examination had shown the patient to have a BRCA1 germline mutation, six courses of docetaxel-cisplatin combination therapy (docetaxel 90 mg/m2 and carboplatin, starting at the area under the curve (AUC) six and later reduced to AUC five) were administered from February to August 2018. Again, this resulted in a complete response, and the patient was started on olaparib 600 mg/day, which is an effective maintenance therapy for recurrent ovarian cancer, especially with the BRCA1 mutation, and shows sensitivity to platinum-based anti-cancer drugs [9]. At the time of writing this case report, the patient was still receiving olaparib therapy without evidence of cancer recurrence (Figure 2).

During this period, the vestibular schwannoma remained stable but began to show a slight increase in size from July 2015 until April 2017 (Figure 1B). After the start of carboplatin and docetaxel chemotherapy, MRI showed a decrease in the volume of the tumor for the first time (Figure 1C). The tumor continued to shrink thereafter and to an even greater extent after the start of olaparib chemotherapy (Figure 1D, Figure 2). Using MRI, the tumor volume was measured on constructive interference in steady-state (CISS) images at each point and compared using a 3D image analysis system, SYNAPSE VINCENT (Fujifilm, Tokyo, Japan). When the patient was first diagnosed in March 2013, the volume was 1.447 ml but had shrunk by 64.5% to 0.514 ml by April 2021.

## Discussion

In this case, although a histological diagnosis of a brain tumor was not achieved, a vestibular schwannoma was the most likely diagnosis based on the location, imaging findings, and disease course. Vestibular schwannomas are known to sometimes regress spontaneously, as 3.81% of those who were adapted to the 'wait and scan' strategy showed shrinkage according to the study in patients registered in a national database in Denmark [10]. Characteristics associated with spontaneous regression include a large lesion size at the time of diagnosis and protrusion beyond the floor of the inner ear canal [11]. However, none of these features were evident in this case.

On the other hand, at the time of regression, chemotherapy had been administered for ovarian cancer, making it highly possible that the chemotherapy was related to the regression of the tumor. Whereas bevacizumab and lapatinib are shown to have some efficacy for tumors associated with NF2, there have been few reports of effective drug treatment for sporadic vestibular schwannomas.

The present patient had a germline mutation of BRCA1, and such patients are more likely to develop cancers such as those of the breast, ovary, prostate, and pancreas. Although only an association between a family history of breast cancer and meningioma risk is known and none for BRCA1 and vestibular schwannoma, the possibility that BRCA1 mutations are involved in the development of vestibular schwannoma cannot be ruled out and may present a way for future research in this area.

Four drugs were used to treat ovarian cancer in this patient, including docetaxel and olaparib, which were newly administered during the period of tumor shrinkage after the tumor had reached its maximum volume in April 2017. These drugs may have played a role in tumor regression. In particular, poly ADP-ribose polymerase-1 (PARP-1), the target of olaparib, usually has a growth-inhibitory role and induces necrosis in DNA-damaged cells, but in some situations, it can influence the Ras-Raf-MEK-ERK pathway (MAPK/ERK) signaling pathway [12]. Even if this tumor had arisen through a mechanism unrelated to BRCA1, it is still possible that olaparib may have suppressed its growth via the phosphatidylinositol-3-kinase (PI3K)/Akt, mammalian target of rapamycin (mTOR), and mitogen-activated protein kinase (MAPK) pathways, because Merlin, which is frequently mutated in many vestibular schwannomas, is a molecule also involved in these pathways [13].

## Conclusions

This is the first report of an acoustic tumor presumed to be a vestibular schwannoma, accompanied by germline mutations of BRCA1, and also the first recorded case of an acoustic tumor showing a decrease in size in response to chemotherapy for ovarian cancer. We believe the present case is very informative when considering future treatment and research options for vestibular schwannoma. Further accumulation of molecular biological data and observations of therapeutic response may reveal future avenues of treatment for vestibular schwannoma.
